# Raging Diseases

**Published:** 1870-11

**Authors:** 


### RAGING DISEASES.

It is reported that chill and fever is prostrating whole communities; that five hundred families are stricken with it in New Haven; that in Poughkeepsie there are not physicians enough to attend the hourly increasing demands for medical aid; that one physician was called to thirty new cases in a single night; that it prevails all over Staten Island, Long Island and New Jersey, and in many places where it has not been known in many years.

Intermittent Fever, Chill and Fever, Fever and Ague, are one and the same disease, and are caused in the same way the world over, by the decomposition of vegetable matter, and can be produced in no other way. Three things must be always present—wood, leaves, fruits, or whatever grows out of the ground: there must be moisture, and there must be a heat of over eighty degrees continued for some days. Whether this decaying vegetation feeds living things animal or vegetable, and they cause the disease, is another question; it is enough for us to know that dead vegetation, moisture and heat combined, will so affect the atmosphere as to give these maladies to communities and individuals who breathe it. Hence, September, when the days are hot and the leaves are falling and decaying in the dampness, is called "The Month Malign," and people are sickening and burning with fever, and shaking with cold alternately, in millions of families, for the want of a little sense, just a mite.

In ponds and bayous, and sluggish streams, vegetation is always decomposing and decaying more or less completely, and the particles thus decayed sink to the bottom, and appear to us in the shape of soft black mud; and whenever that mud is exposed to a hot sun, certain particles arise with a nature so malignant, that filling the air, they are breathed into the lungs, and cause all the fevers from the mildest intermittent to the black vomit.

Yellow fever is nothing more or less than fever and ague intensified.

Here is a beautiful lake or extensive mill-pond, but intermittents have never been known there. Drain that pond in the dog-days, so that the sun can shine on its black, slimy, muddy bottom, and in less than a week the whole community will be prostrated with fevers, growing more and more malignant, until the congestive chill will kill in an hour. Cover every inch of that muddy surface with water a foot in depth, and the malady will begin to abate in twenty-four hours, because the deadly particles cannot rise through that water; and if it be a running stream, the abatement will be more striking, because running water attracts and absorbs the peccant material called "Miasm," meaning an emanation, from its rising up from the ground. Malaria means simply bad air; any impure air is malaria, whether impregnated with the odor of the rose or assafœtida, so miasm is malaria, a bad air; but malaria is not necessarily miasm; it must have decayed vegetation in it to make it miasm. So intermittents depend on the amount of marsh bottom exposed to the sun in dog-days, because then the sun is hot.

Whenever there is a greater heat or a greater drought than common, a larger amount of marsh bottom is exposed to the sun, and the lower the ponds are on the rivers, or places of standing water, the greater will be the sickness; the speediest remedy is to fill up these ponds to the highest water-mark; but to make the place permanently healthy, drain the ponds in mid-winter, and in the spring fill them up with earth, or prevent any more water from going into them.

From the principles already laid down, heavy rains will make a sickly place healthy by covering all bottoms with water, and for the same reason heavy rains may make a healthy locality sickly when the sun comes out, because the water is soon evaporated from the many indentations in fields and roadways, each indentation leaving its damp bed exposed to the sun.

When intermittents prevail, sleep with all the outer windows and doors closed from half an hour before sundown until half an hour after sunrise; get your breakfast before sunrise, and your supper before sunset, eating them both by a blazing fire, not going outside the door in the morning until after breakfast. These precautions will counteract fever and ague in all localities, on principles often laid down at length in former years in the pages of the *Journal.*

